# Impaired arterial dilation and increased NOX2 generated oxidative stress in subjects with ataxia-telangiectasia mutated (ATM) kinase

**DOI:** 10.1016/j.redox.2024.103347

**Published:** 2024-09-12

**Authors:** Lorenzo Loffredo, Annarosa Soresina, Bianca Laura Cinicola, Martina Capponi, Francesca Salvatori, Simona Bartimoccia, Vittorio Picchio, Maurizio Forte, Caterina Caputi, Roberto Poscia, Vincenzo Leuzzi, Alberto Spalice, Pasquale Pignatelli, Raffaele Badolato, Marzia Duse, Francesco Violi, Roberto Carnevale, Anna Maria Zicari, AT study group, Ilaria Maria Palumbo, Arianna Magna, Alessia Fallarino, Arianna Pannunzio, Enrico Maggio, Chiara Bagnato, Vittoria Cammisotto, Valentina Castellani

**Affiliations:** i Department of Clinical, Internal, Anesthesiologic and Cardiovascular Sciences, Sapienza University of Rome, Italy; j Department of General Surgery and Surgical Specialty, Sapienza University of Rome, Rome, Italy; k Department of Translational and Precision Medicine, Sapienza University of Rome, Rome, Italy; a Department of Clinical, Internal, Anesthesiologic and Cardiovascular Sciences, Sapienza University of Rome, Italy; b Pediatrics Clinic and Institute for Molecular Medicine A. Nocivelli, Department of Clinical and Experimental Sciences, University of Brescia and ASST-Spedali Civili di Brescia, Brescia, Italy; c Department of Maternal, Child Health and Urological Sciences, Sapienza University, Rome, Italy; d Department of Medical-Surgical Sciences and Biotechnologies, Sapienza University of Rome, Latina, Italy; e IRCCS Neuromed, Pozzilli (IS), Italy; f Department of Human Neuroscience - Unit of Child Neurology and Psychiatry, Sapienza University of Rome, Rome, Italy; g Clinical Research Unit, AOU Policlinico Umberto I- Sapienza University of Rome, Rome, Italy; h Sapienza University of Rome, Rome, Italy

## Abstract

**Background:**

Subjects with mutations in the Ataxia-Telangiectasia mutated (ATM) gene encoding for ATM kinase have a greater predisposition to develop atherosclerosis, but the mechanism behind this phenomenon is not yet understood. NADPH oxidase type 2 may play a role in this process, leading to endothelial dysfunction and an increased susceptibility to thrombosis. The purpose of this study was to assess the redox state in individuals with ATM mutations and determine its impact on endothelial function.

**Methods:**

In this cross-sectional study, twenty-seven children with ataxia telangiectasia (AT) (13 males and 14 females, mean age 15.1 ± 7.6 years) were compared with 27 controls (13 males and 14 females, mean age 14.6 ± 8.4 years) matched for age and gender. Additionally, 29 AT parents with heterozygous mutation of ATM (h-ATM) gene, and 29 age- and gender-matched controls were included. Endothelial function was evaluated through brachial flow-mediated dilation (FMD) and the assessment of nitric oxide (NO) bioavailability. Oxidative stress was evaluated by measuring serum activity of soluble NOX2-dp (sNOX2-dp), hydrogen peroxide (H_2_O_2_) production, and hydrogen breakdown activity (HBA). Thrombus formation was assessed through the Total Thrombus Formation Analysis System (T-TAS).

**Results:**

AT children and parents with heterozygous ATM mutations exhibited significantly lower FMD, HBA, and NO bioavailability as compared to age and gender matched controls. AT children and ATM carrier of heterozygous ATM mutations had significantly higher concentrations of sNOX2-dp and H_2_O_2_ as compared to controls. Compared to the respective controls, AT children and their parents, who carried heterozygous ATM mutation, showed an accelerated thrombus growth as revealed by reduced occlusion time. Multivariable linear regression analysis revealed that sNOX2 (standardized coefficient β: −0.296; SE: 0.044; p = 0.002) and NO bioavailability (standardized coefficient β: 0.224; SE: 0.065; p = 0.02) emerged as the only independent predictive variables associated with FMD (R^2^: 0.44).

**Conclusions:**

This study demonstrates that individuals with ATM mutations experience endothelial dysfunction, increased oxidative stress, and elevated thrombus formation. These factors collectively contribute to the heightened susceptibility of these individuals to develop atherosclerosis.

## Graphical abstract

**Figure:**
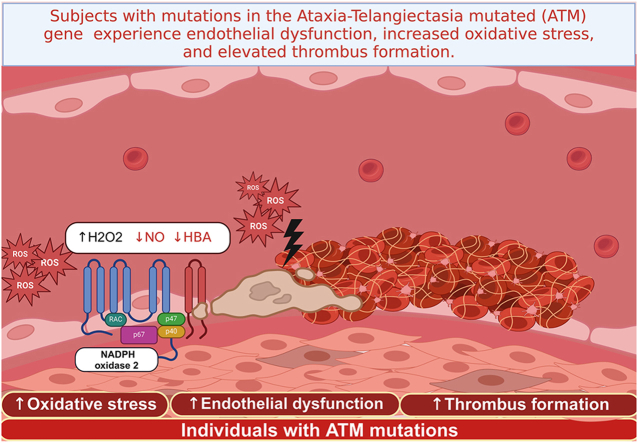

## 1 Introduction

Cardiovascular diseases are the leading cause of mortality worldwide, necessitating a comprehensive understanding of the intricate molecular mechanisms that underlie vascular health and dysfunction. In recent years, substantial progress has been made in unraveling the roles of key molecular players involved in vascular homeostasis and perturbation. Among these, the ataxia-telangiectasia mutated (ATM) kinase and nicotinamide adenine dinucleotide phosphate (NADPH) oxidase (Nox) emerged as critical elements that contribute to the delicate balance between vascular health and disease progression [1,2].

The ATM protein is the product of the ATM gene, located on chromosome 11q22-23 [3]. ATM is a member of the family of phosphoinositide 3-kinase-related kinases, and its activation is regulated by DNA damage-dependent mechanisms (double-strand DNA breaks) or reactive oxygen species (ROS)-dependent mechanisms [4]. ATM is recruited to sites of double-strand DNA breaks by the MRE11–RAD50–NBS1protein-complex (MRN) [4]. Subsequently, ATM undergoes autophosphorylation and acetylation [4]. The association of ATM with MRN promotes ATM monomerization and enhances the stable binding of substrates [4]. Additionally, ATM protein can be activated independently of DNA and MRN through oxidative stress, which leads to the formation of intermolecular disulfide bonds, regulating ROS formation [4].

ATM, has garnered significant attention for its multifaceted roles beyond genomic stability [4,5]. Recent studies have highlighted ATM's involvement in several cellular processes, including oxidative stress response, redox signaling, autophagy and inflammation [4,6]. ATM plays a role in modulating NOX activity, a pivotal source of ROS production in white blood cells [7,8]. In particular, in phagocytes, phosphorylation by ATM kinase down-regulates NOX2 activity [7]. Conversely, the oxidative stress generated by NOX2 following various stimuli (e.g. lipopolysaccharides (LPS) and ionizing radiation) activates ATM kinase to counterbalance the excess of ROS [9].

Ataxia telangiectasia (AT), originally known as Louis-Bar Syndrome, is a rare autosomal recessive condition (prevalence: 40,000–1:300,000) [10], due to homozygous mutation of ATM gene associated to inability to protect cells from an excess of oxidative stress that will lead to an increased risk in the development of tumors and of cardiovascular diseases [1]. Additionally, the frequency of ATM mutations carriers in the general population ranges from 1.4 % to 2 % [10]. Individuals with ATM mutations are at increased risk of developing atherosclerosis, diabetes mellitus [3], heart failure [11], and coronary heart disease [1]. The specific mechanisms underlying this increased cardiovascular risk are yet to be fully elucidated.

Flow-mediated dilatation (FMD) of the brachial artery is considered a marker of subclinical atherosclerosis and endothelial function [12,13]. FMD is influenced by redox status as it is dependent upon endothelial biosynthesis of nitric oxide (NO), a powerful vasodilator molecule with antioxidant property [14]. Impaired FMD is detectable in patients at risk or with cardiovascular disease and reversed by antioxidant infusion in human [15]; also lowered FMD is associated with enhanced risk of cardiovascular events [14,16]. Arterial vasodilation is closely linked to the activation of NOX2, as shown by chronic granulomatous disease (CGD), that is characterized by hereditary deficiency of NOX2, limited or no production of oxygen free radicals and a notable increase in FMD [12,17,18]. Considering the closing interplay between AT and NOX2, we speculate that patients with ATM mutation may be featured by NOX2 overactivation and impaired FMD. To explore this hypothesis we evaluated the redox status of individuals with ATM kinase mutations and carriers of ATM kinase mutation and its impact on endothelial function.

## 2 Methods

Twenty-seven children with AT, carrying homozygous mutation of the ATM gene, and 27 controls matched for age and gender were recruited between 2019 and 2023; furthermore, 29 AT parents, with heterozygous mutation of the ATM gene, and 29 age and gender matched controls were recruited.

Molecular genetic testing was utilized for diagnosing Ataxia-Telangiectasia in both homozygous and heterozygous forms, employing targeted sequencing of the ATM gene situated on chromosome 11q22-23 [3].

AT children were excluded from the study if they had one of the following characteristics: acute infection, obesity or severely underweight, active smoking, cardiopulmonary diseases, severe nephropathy and liver disease.

Hypercholesterolemia and diabetes were defined for adults and children according to the previous published guidelines [19,20].

A cross-sectional study was performed to compare:

- endothelial function assessed by FMD and NO bioavailability;
  - oxidative stress by assessment of serum activity of soluble NOX2-dp, hydrogen peroxide (H_2_O_2_) production and hydrogen break-down activity (HBA);
- thrombus formation evaluated by Total Thrombus formation Analysis System (T-TAS).

Written informed consent was obtained from all participants (or from parents if the subject was a minor) according to Italian regulations. The experimental procedure was approved by the Institutional Review Board at Sapienza University of Rome (ref. no. 5661) and was conducted in accordance with the Declaration of Helsinki.

### 2.1 Blood sampling and preparations

Blood samples were collected between 8 and 9 a.m. from the antecubital vein in fasting conditions and collected in BD Vacutainer with or without anticoagulants (trisodium citrate, 3.8 %, 1/10 (v/v) or 7.2 EDTA). Blood anticoagulated with sodium citrated (3.8 %, 1/10 (v: v)) was centrifuged for 15min at 180g at room temperature (RT) and Platelet-Rich Plasma (PRP, 2 × 105 platelets/μL) was prepared as previously described [21].

Blood without anticoagulants was centrifuged at 300×*g* for 10 min at room temperature (RT). Serum and plasma samples were separated into aliquots and stored at −80 °C until analyses.

### 2.2 Brachial FMD

Ultrasound assessment of basal brachial diameter and endothelial dependent FMD of brachial artery were investigated according to the guidelines [22] and as previously described [23].

A 7.5-MHz linear array transducer ultrasound system (Samsung HS30, Samsung, Seoul, Korea) equipped with electronic callipers was used to measure the brachial FMD.

### 2.3 Nitric oxide assay

Nitric oxide (NO) was evaluated in serum by NO^2−^/NO^3−^ determination. Briefly, the nitrate (NO^3−^) in the sample is converted into nitrite (NO^2−^) by nitrate reductase enzyme, and then total nitrite is detected with Griess Reagents as a coloured azo dye product (absorbance 540 nm). Values were expressed as μM. Intra- and inter-assay coefficients of variation were <10 %.

### 2.4 sNOX2-dp assay

NOX2 activity was measured in serum as soluble NOX2 derived peptide (sNOX2-dp) with a previously described ELISA method [24]. Values were expressed as pg/mL and both intra- and inter-assay coefficients of variation were <10 %.

### 2.5 H_2_O_2_ production

Hydrogen peroxide (H_2_O_2_) was measured by a colorimetric assay as described previously [25]. The final product was read at 450 nm and expressed as μM. Intra- and inter-assay coefficients of variation were both <10 %.

### 2.6 Serum hydrogen peroxide scavenging activity

Hydrogen peroxide (H_2_O_2_) break-down activity by HBA assay kit (Aurogene, code HPSA-50) was used to measure the antioxidant capacity of serum samples. The % of HBA was calculated according to the following formula: % of HBA = [(Ac − As)/Ac] × 100, where Ac is the absorbance of H_2_O_2_ and As is the absorbance in the presence of the serum sample.

### 2.7 Thrombus formation

Thrombus growth under flow conditions was measured by thrombus-formation analysis system (T-TAS®01 apparatus, Fujimori Kogyo Co., Ltd., Japan) on PL-chips. Whole blood (400 μL) anticoagulated by BAPA (benzylsulfonyl-D-argininyl-prolyl-4-amidinobenzylamide) from AT children, AT parents and respective controls were collected. Then, 340 μL of samples were transferred to the PL-chip and analyzed. Growth, intensity, and stability of the formation of platelet clots were measured by the time needed to reach the occlusion pressure (occlusion time), and the area under the flow-pressure curve (AUC) parameter, that is an area under the pressure curve from the start of the test to a time of 10 min, were studied [26].

### 2.8 Statistical analysis

Continuous variables were expressed as mean ± standard deviation and categorical variables as percentage. To evaluate if variables have a normal distribution a Shapiro-Wilk test was executed. Differences between percentages were analyzed by the chi-square test. The analysis of differences between groups was obtained with T-test for variables normally distributed and with non-parametric tests (Mann–Whitney *U* test) for those not-normally distributed. The Spearman correlation test was used for bivariate analysis; the variables with evidence of an association with p < 0.10 were included in a multivariable linear regression analysis using a stepwise procedure. P < 0.05 was considered as statistically significant. All analyses were carried out with SPSS (IBM SPSS Statistics V.25.0) and with GraphPad Prism 7 (GraphPad Software La Jolla, CA 92037 USA).

Sample size calculation was computed with respect to a two-tailed Student's t-test for independent groups, considering: 2.5 % (δ) as difference for FMD between AT children and controls, 2.5 % as SD, 0.05 (α) as type I error probability and 0.95 as power 1−β. The minimum sample size was 26 patients per group.

## 3 Results

Twenty-seven AT patients (13 males and 14 females, mean age 15.1 ± 7.6 years) and 27 controls (13 males and 14 females, mean age 14.6 ± 8.4 years) were recruited. In addition, twenty-nine AT parents with heterozygous mutation of ataxia-telangiectasia(10 males and 19 females, mean age 48.6 ± 8.4 years) and controls (13 males and 14 females, mean age 49.9 ± 7.3 years) were enrolled. Clinical characteristics of these 4 groups are described in Table 1. No statistically significant differences were found among the major cardiovascular risk factors in the study groups. Five patients with AT had a history of neoplasms (including Burkitt's lymphoma, osteosarcoma, astrocytoma, acoustic neurinoma, and ameloblastoma). No significant difference was observed among the groups concerning fasting glycaemia or hypercholesterolemia.

**Table 1:** Clinical characteristics of the study population. Continuous data are expressed as mean values ± standard deviation. The statistical comparison between the groups was carried out with the T-test or when reported (∗) with the Mann-Whitney test.

	AT	AT Controls	p value	Individuals with heterozygous mutation of ATM gene	Controls of individuals with heterozygous mutation of ATM gene	p value –
**N.**	27	27	–	29	29	
Age (yy)	15.1 ± 7.6	14.6 ± 8.4	0.801	48.6 ± 8.4	49.9 ± 7.3	0.520
Gender (M/F)	13/14	13/14	1.0	10/19	10/19	1.0
BMI (Kg/m2)	16.6 ± 3.2	16.9 ± 2.7	0.772	25.7 ± 3.6	25.0 ± 2.8	0.431
Systolic BP (mmHg)	105 ± 8	106 ± 7	0.754	126 ± 20	121 ± 11	0.261
Diastolic BP (mmHg)	65 ± 8	66 ± 6	0.672	80 ± 10	79 ± 8	0.626
Total cholesterol mg/dl	137 ± 24	134 ± 28	0.677	130 ± 17	128 ± 21	0.709
Glycaemia (mg/dl)	85 ± 9	84 ± 8	0.715	90 ± 9	87 ± 7	0.201
Kidney failure	0	0	–	0	0	–
Hypertension	0	0	–	5	5	1.0
Previous Smoking	0	0	–	4	5	0.717
Hypercholesterolemia	2	0	0.150	2/29	2	1.0
Diabetes	1	0	0.313	0	0	–
**Medications**						
Intra Erythrocyte Dexamethasone	7	0	<0.001	0	0	–
Intravenous or sub-cutaneous immunoglobulins	10	0	<0.001	0	0	–
Statins	1	0	0.313	0	0	–
Metformin	1	0	0.313	0	0	–
Antihypertensive drugs	0	0	–	4/29	5	0.716
Calcium oral intake	6	0	<0.001	0	0	–
Iron oral intake	2	0	0.150	0	0	–
N-Acetil-Cisteine	3	0	0.075	0	0	–
Carnitine	1	0	0.313	0	0	–
trimethoprim and sulphamethaoxazole	14	0	<0.001	0	0	–
FMD (%)	2.1 ± 3.1	8.5 ± 4.2	<0.001∗	4.6 ± 3.7	7.2 ± 2.5	0.003∗
NO bioavailability (μM)	10.5 ± 6.0	18.7 ± 5.2	<0.001∗	16.8 ± 3.6	20.8 ± 4.4	<0.001∗
sNOX2-dp (pg/ml)	29.1 ± 9.8	14.5 ± 3.6	<0.001	16.7 ± 5.5	12.2 ± 4.2	0.001
H_2_O_2_ (μM)	25.8 ± 8.6	14.8 ± 4.3	<0.001∗	15.9 ± 5.0	12.1 ± 4.2	0.01∗
HBA (%)	30.0 ± 11.0	43.6 ± 12.9	<0.001	41.0 ± 9.7	50.2 ± 13.5	<0.001
Occlusion Time (s)	349 **±** 65	453 **±** 42	<0.001∗	433 **±** 77	488 **±** 58	0.003
AUC (%)	366 **±** 50	246 **±** 48	<0.001	262 **±** 70	210 **±** 58	0.004

**Fig. 1:**
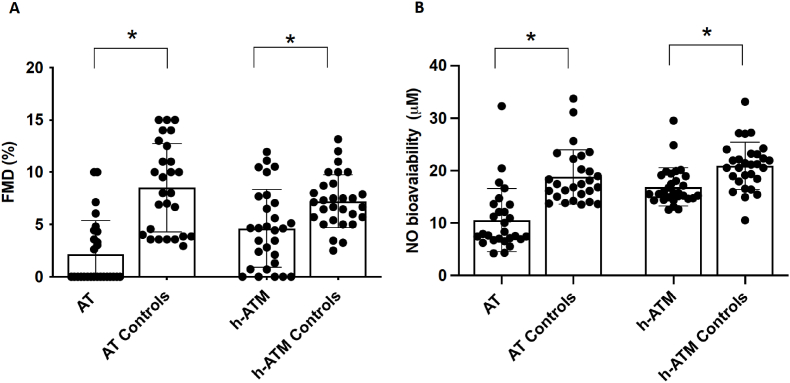
FMD measurement (Panel A), NO bioavailability (Panel B) in AT children (n = 27), individuals with heterozygous mutation of ATM (h-ATM) gene (n = 29), children (AT Controls n = 27) and adult controls (h-ATM Controls n = 29). Data are expressed as mean values ± standard deviation (SD), and ∗p < 0.01.

Compared to the respective controls, FMD and NO bioavailability were significantly lower in AT children and in parents with carriers of heterozygous ATM mutation(Table 1 and Fig. 1A and B); of note, FMD was reduced by roughly 75 % and 36 % in homozygous and heterozygous subjects respectively.

**Fig. 2:**
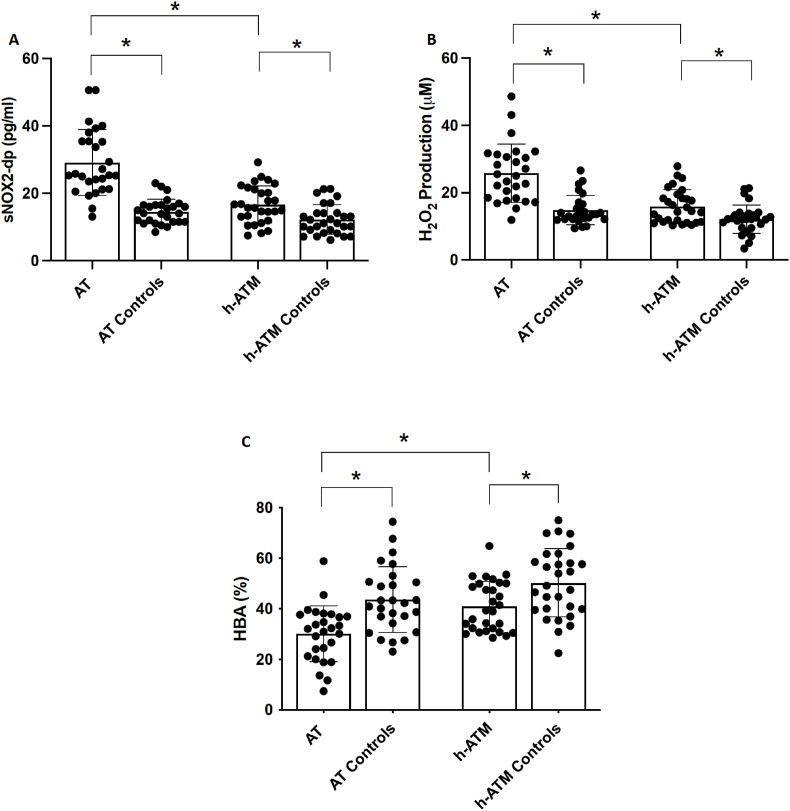
sNOX2-dp (Panel A), H_2_O_2_ (Panel B), HBA (Panel C) in AT children (n = 27), individuals with heterozygous mutation of ATM (h-ATM) gene (n = 29), children (AT Controls n = 27) and adult controls (h-ATM Controls n = 29). Data are expressed as mean values ± standard deviation (SD), and ∗p < 0.01.

Both groups of AT children and ATM mutation carriers had significantly higher blood concentration of sNOX2-dp and H_2_O_2_ in comparison to control subjects (Table 1, Fig. 2A and B) (Table 1 and Fig. 2A and B). Conversely, blood HBA was significantly lower in both AT subjects and in ATM carriers in comparison to control subjects (Table 1 and Fig. 2C).

**Fig. 3:**
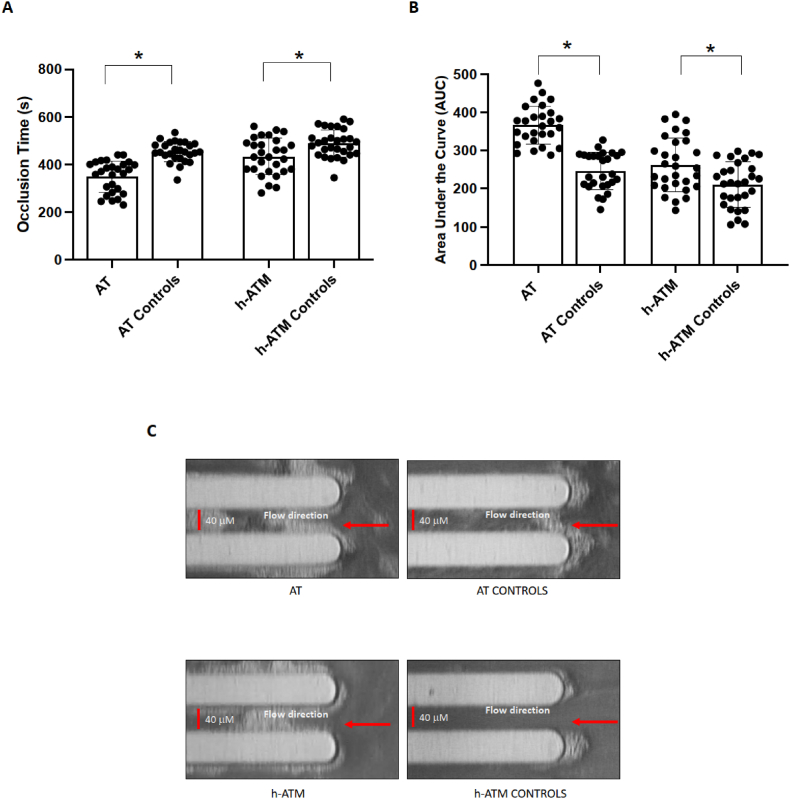
Parameters of thrombus formation, Occlusion Time (Panel A) and area under the curve (AUC) (Panel B), in AT children (n = 27), individuals with heterozygous mutation of ATM (h-ATM) gene (n = 29), children (AT Controls n = 27) and adult controls (h-ATM Controls n = 29). Data are expressed as mean values ± standard deviation (SD), and ∗p < 0.01. Representative picture of thrombus formation (Panel C) in AT children, individuals with heterozygous mutation of ATM (h-ATM) gene, children, and adult controls. Blood flow direction is from right to left (see arrow). The width and depth of the capillaries are both 40 μm.

Furthermore, we evaluated thrombus formation by T-TAS analysis, in AT children, in ATM mutations carriers and in control groups. Compared to the respective controls, AT children and their parents, who carried heterozygous ATM mutation, show an accelerated thrombus growth as revealed by reduced occlusion time and increased AUC (Table 1, Fig. 3A–C).

**Fig. 4:**
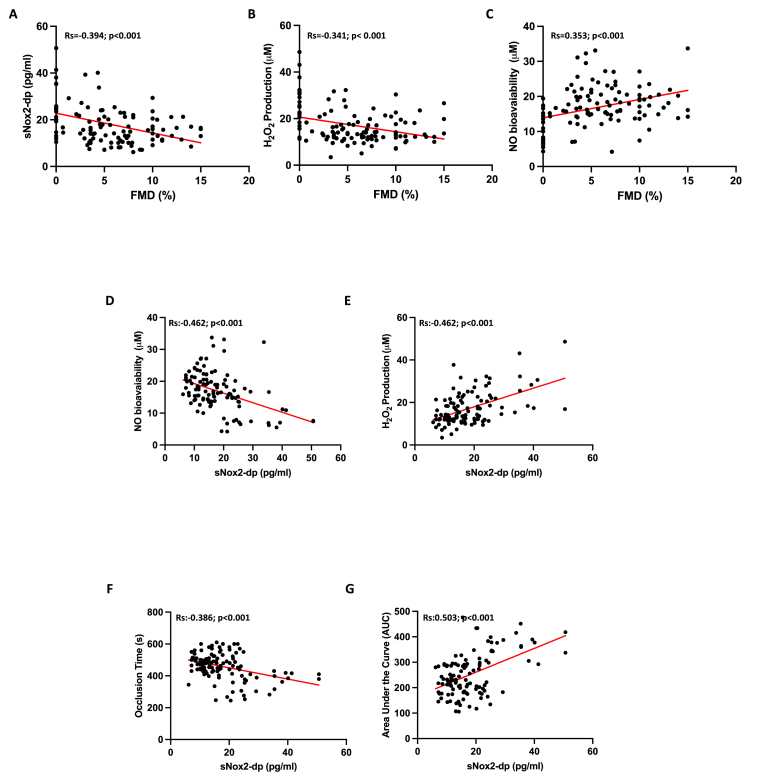
Correlation analysis between FMD and sNOX2-dp (Panel A), H_2_O_2_ (Panel B), NO bioavailability (Panel C) and between sNOX2-dp and NO bioavailability (Panel D), H_2_O_2_ (Panel E), Occlusion Time (Panel F) and Area Under the Curve (AUC) (Panel G).

The bivariate analysis revealed significant correlations: FMD was associated with sNOX2-dp (Rs = −0.394, p < 0.001) (Fig. 4, Panel A), H₂O₂ (Rs = −0.341, p < 0.001) (Fig. 4, Panel B), and NO bioavailability (Rs = 0.353, p < 0.001) (Fig. 4, Panel C). Additionally, sNOX2-dp correlated with NO bioavailability (R = −0.462, p < 0.001) (Fig. 4, Panel D), H₂O₂ (R = 0.512, p < 0.001) (Fig. 4, Panel E), OT (R = −0.386, p < 0.001) (Fig. 4, Panel F), and AUC (R = 0.503, p < 0.001) (Fig. 4, Panels A–G). No linear correlation was found between FMD, sNOX2-dp, H2O2, NO and HBA with cholesterol and blood glucose.

Multivariable linear regression analysis showed that sNOX2-dp (standardized coefﬁcient β: 0.296; SE: 0.044; p = 0.002) and NO (standardized coefﬁcient β:0.224; SE: 0.065; p = 0.02) emerged as the only independent predictive variables associated with FMD (R^2^:0.44).

## 4 Discussion

The study reveals that individuals with homozygous ATM gene mutations, affected by AT, and their parents, who carry heterozygous ATM mutations, show endothelial dysfunction, increased NOX2 activation and impaired antioxidant activity (Central illustration). Children with homozygous mutations exhibited two fold higher oxidative stress values, halved antioxidant status, and four times lower endothelial function compared to controls. Similar findings were detected in heterozygous ATM mutation carriers with differences, that were less marked in terms of reduced FMD and oxidative stress compared to the 29 respective controls, suggesting distinct phenotypes dependently upon gene activation patterns.

Subjects with AT mutations have shown an elevated risk to develop atherosclerosis and its sequelae as shown by Su [27] and Swift [28] who observed elevated mortality risk for coronary heart diseases in subjects with ATM heterozygous deficiency. However, the underlying pathogenetic mechanism remains only partially understood. Studies conducted in mouse models with ATM mutations have demonstrated a high susceptibility to insulin resistance [29] and hypercholesterolemia [30], which are the two cardiovascular risk factors that could potentially account for the increased predisposition to atherosclerosis. Specifically, Miles demonstrated that AT mice maintain normal insulin sensitivity but experience transient hyperglycemia during an oral glucose tolerance test [29]; Wu found that heterozygous mutation of the ataxia-telangiectasia mutated gene exacerbates hypercholesterolemia in apoE-deficient mice [30].

This finding raises an important issue as to whether ATM lowers FMD via a metabolic disease-associated arterial dysfunction. Compared to previous human studies reporting either hypercholesterolemia or diabetes in AT patients [31,32], we did not find these metabolic features. This difference may be explained by the lower mean age of our cohort compared to the previous ones and by the fact that age conditions the appearance of metabolic disease, that, in fact, occurs prevalently after the pubertal period [33,34]. Hence, the reduction of FMD in our ATM cohort cannot be attributed to a coexistent metabolic disease affecting arterial dysfunction, that, thereby, must be explained by an alternative mechanism. This hypothesis is also supported by experiments performed in ATM heterozygosis subjects [29], in whom we report for the first time FMD lowering; it is noteworthy that our ATM carrier cohort did not display changes of lipid or glycemic profile that is in agreement with a previous report on the same topic [35].

Previous studies in animal models have shown that the ATM gene regulates oxidative stress by protecting against damage through a reduction in the activity of NOX-2 [7,36]. Thus, the increase of NOX-2 derived ROS formation may shed new light in the putative relationship between mutation of the ATM gene and risk of cardiovascular disease [36,37]. Thus, NOX2 upregulation results in the overproduction of ROS and the inactivation of NO [[38], [39], [40]], both of which are crucial factors in reducing FMD [12]. Accordingly, ATM patients as well as ATM carriers display NOX2 overactivation along with NO lowering. We must recognize, however, that NO blood analysis has intrinsic bias related to various endogenous and exogenous factors, including dietary nitrate uptake, inhalation of atmospheric nitrogen oxides, salivary formation, and renal function [41]. Notably, ATM kinase-related oxidative stress represents the counterpart of chronic granulomatous disease, where impaired NOX2 activation leads to reduced oxidant species production and increased vasodilation [12,17,18,42].

In this study, we not only reveal endothelial dysfunction in subjects with AT gene mutations but also highlight an increased risk of thrombosis, assessed using T-TAS. This susceptibility to thrombosis, previously reported in AT patients as a notable frequency of cerebral thrombosis [43], may result from NOX-2 related clotting and platelet activation [2]; further study is, however, necessary to deeper analyze the effect of ATM on the two systems.

The study has some limitations. The limited sample requires further confirmation with a larger number of homozygous and heterozygous AT subjects. No other sources of oxidative stress from other NADPH oxidase isoforms have been evaluated. Additionally, the lack of data due to the limited sample size regarding the relationship between genetic variations in ataxia-telangiectasia, oxidative stress, endothelial dysfunction, and markers of platelet activation represents another limitation of the study.

In conclusion, this study sheds light on the intricate relationship among endothelial dysfunction, oxidative stress, and individuals carrying mutations in the ATM gene. Given the substantial prevalence of heterozygous individuals for A-T, comprising about 2 % of the adult population [44], the findings of this study suggest that genetic analysis of ATM gene could be particularly valuable in subjects with atherosclerotic disease but without classic cardiovascular risk factors, or in cardiovascular diseases with unclear etiology, to implement patient-targeted prevention strategies.

## Funding information

This study was supported by Sapienza University of Rome to LL by Sapienza University of Rome (grant 2020 Prot. RM1221816616C410. “IDENTIFICATION OF NEW MARKERS TO PREVENT CARDIOVASCULAR DISEASE IN SUBJECTS WITH MUTATIONS IN THE ATM (ATAXIA TELANGIECTASIA, MUTATED) GENE”. PNRR-MR1-2022-12376594 to RB by Ministero della Salute.

## CRediT authorship contribution statement

**Lorenzo Loffredo:** Writing – review & editing, Writing – original draft, Supervision, Methodology, Investigation, Funding acquisition, Formal analysis, Data curation, Conceptualization. **Annarosa Soresina:** Investigation. **Bianca Laura Cinicola:** Investigation, Data curation. **Martina Capponi:** Investigation, Data curation. **Francesca Salvatori:** Data curation. **Simona Bartimoccia:** Methodology, Investigation, Data curation. **Vittorio Picchio:** Methodology, Investigation. **Maurizio Forte:** Methodology, Investigation. **Caterina Caputi:** Investigation. **Roberto Poscia:** Supervision. **Vincenzo Leuzzi:** Supervision, Methodology. **Alberto Spalice:** Supervision. **Pasquale Pignatelli:** Visualization, Supervision, Methodology. **Raffaele Badolato:** Writing – review & editing, Supervision, Methodology. **Marzia Duse:** Writing – review & editing, Supervision, Conceptualization. **Francesco Violi:** Writing – review & editing. **Roberto Carnevale:** Writing – review & editing, Methodology, Investigation. **Anna Maria Zicari:** Writing – review & editing, Supervision, Methodology, Investigation, Data curation, Conceptualization. **Ilaria Maria Palumbo:** Investigation. **Arianna Magna:** Investigation. **Alessia Fallarino:** Investigation. **Arianna Pannunzio:** Investigation. **Enrico Maggio:** Investigation. **Chiara Bagnato:** Investigation. **Vittoria Cammisotto:** Investigation. **Valentina Castellani:** Investigation.

## Declaration of competing interest

The authors declare no conflict of interest.

## Data Availability

Data will be made available on request.

## Appendix

AT study group is composed of:

Ilaria Maria Palumbo MD^1^, Arianna Magna MD^1^, Alessia Fallarino MD^1^, Arianna Pannunzio MD^1^, Enrico Maggio MD^1^, Chiara Bagnato MD^1^, Vittoria Cammisotto PhD^1^, Valentina Castellani PhD^2^

^1^Department of Clinical, Internal, Anesthesiologic and Cardiovascular Sciences, Sapienza University of Rome, Italy

^2^Department of Medical-Surgical Sciences and Biotechnologies, Sapienza University of Rome, Latina, Italy
